# Shingles vaccination before targeted therapy: why clear guidance isn’t enough

**DOI:** 10.1093/rap/rkag047

**Published:** 2026-05-04

**Authors:** Hannah Kim, Nilay Joshi, Sara Naqvi, Ania Manson, James Galloway

**Affiliations:** Department of Rheumatology, Kettering General Hospital NHS Foundation Trust, Kettering, UK; Department of Rheumatology, Kettering General Hospital NHS Foundation Trust, Kettering, UK; Department of Rheumatology, Kettering General Hospital NHS Foundation Trust, Kettering, UK; Department of Clinical Immunology, Cambridge University Hospitals NHS Foundation Trust, Cambridge, UK; Department of Rheumatology, King’s College Hospital NHS Foundation Trust, London, UK; Centre for Rheumatic Diseases, King’s College London, London, UK

## Introduction

Varicella zoster virus (VZV) reactivation poses a genuine threat to patients receiving immunosuppressive therapy for inflammatory arthritis, particularly with Janus kinase inhibitors or other targeted agents that impair antiviral immunity, including anifrolumab [1, 2]. UK guidance now recommends vaccination with Shingrix (GlaxoSmithKline)—a highly effective recombinant (non-live) zoster vaccine—for all severely immunosuppressed adults ≥18 years of age [3]. Yet practice remains inconsistent. A small UK survey of rheumatologists and immunologists illustrates this gap, highlighting that even clear national guidance does not automatically translate into uniform practice.

Between June and November 2025, a cross-sectional online survey explored approaches to VZV screening and vaccination before initiating biologic or targeted synthetic DMARDs. Disseminated via professional networks and word of mouth, the survey explored antibody testing and preferred management across clinical scenarios varying by patient age, serostatus (including equivocal titres) and presence or absence of vaccination contraindications. Fifty-one clinicians responded, predominantly from rheumatology (76%), with contributions from immunology (10%) and allied professionals including speciality trainee (registrar) doctors, specialist nurses and pharmacists. Most (80%) reported routinely testing serostatus irrespective of self-reported exposure history.

## Key findings

Responses showed broadly consistent, age-stratified patterns for straightforward scenarios but marked heterogeneity in complex situations (Table 1). For younger patients with equivocal titres, responses were almost evenly divided between offering Shingrix, offering varicella vaccination, seeking specialist advice and deferring vaccination altogether. Only 23% reported having formal local guidelines for VZV screening and vaccination, highlighting an infrastructure gap at the service level, although these findings derive from a small, self-selected sample.

**Table 1:** Survey responses by clinical scenario.

Scenario	Preferred approach
Non-immune, no contraindications, age 18–49 years	Varilrix/Varivax preferred; 22% would offer Shingrix
Non-immune, no contraindications, age ≥50 years	Shingrix preferred; 31% would offer Varilrix/Varivax
Equivocal titre, age 18–49 years	No consensus: Shingrix (24%), Varilrix/Varivax (22%), seek specialist advice (16%), defer vaccination (24%)
Equivocal titre, age ≥50 years	Shingrix preferred (61%)
Already on immunosuppression, age 18–49 years	Shingrix preferred (35%); 22% would seek specialist advice
Already on immunosuppression, age ≥50 years	Shingrix preferred (51%); 24% would seek specialist advice

Free-text comments reinforced these themes. Some reported using Shingrix off licence in younger adults or developing local protocols, whereas others expressed concern about equivocal titres and the reliability of serology in immunocompromised individuals. Overall, respondents were clearly engaged but lacked consistent frameworks for decision-making, particularly in borderline scenarios.

## What the guidelines now say

Updated Joint Committee on Vaccination and Immunisation (JCVI) recommendations (November 2024) and subsequent implementation guidance (July 2025), with expanded eligibility from September 2025, have substantially clarified national policy. Shingrix is now recommended for all severely immunosuppressed adults ≥18 years of age, with no upper age limit, and previous contraindications for patients already on immunosuppressive therapy have been removed [3].

These updates address several uncertainties identified in the survey. However, because the survey captured practice both before and shortly after these changes, it cannot reliably assess their impact on clinical behaviour.

## Why guidelines alone are insufficient

VZV vaccination often sits at the periphery of rheumatology practice. In busy clinics, disease control, treatment escalation and monitoring for drug toxicity understandably take precedence. Preventive interventions falling outside routine workflows may be easily deferred or overlooked, failing to embed into routine practice [4].

A central challenge is the division of responsibility between secondary and primary care. Rheumatologists typically identify the need for vaccination when planning targeted therapy, but general practitioners (GPs) hold vaccine stock, run call–recall systems and administer vaccines. Unless responsibilities are explicit, patients may fall into the space between decision and delivery.

Clinic letters may not clearly state that patients meet eligibility criteria for Shingrix, and GPs may not automatically link a new DMARD with the expanded shingles program. Updated guidance assigns GPs responsibility for calling and recalling eligible cohorts [5], but this assumes they know which patients are severely immunosuppressed—a gap that only reliable, timely communication from secondary care can bridge.

System-level pressures compound this. UK rheumatology services are operating under sustained strain and workforce shortfalls, with demand outstripping capacity [6]. The modest survey response rate, despite wide circulation, likely reflects this reality. Clinicians under pressure have limited bandwidth to respond to surveys, absorb new guidance or implement recommendations not perceived as immediately critical. In such circumstances, preventive tasks that lie outside core disease management are among the first casualties.

## Limitations

A key limitation was that survey data collection occurred shortly before and after the publication of updated recommendations. Awareness of these changes among respondents upon survey completion cannot be assumed, therefore this temporal context must be considered when interpreting the findings. The sample size was small and likely biased towards more engaged clinicians with sufficient interest and time to respond. If substantial variation was evident even within this motivated cohort, uncertainty in the wider clinical community is possible. The low response rate may itself indicate VZV vaccination is not currently a salient priority. Consequently, our findings may underestimate rather than overestimate the implementation gap.

## A practical approach

Considering the updated JCVI recommendations, a pragmatic approach for clinicians initiating targeted therapies is as follows:

- Offer Shingrix to adults starting or already on targeted therapies who meet criteria for severe immunosuppression, regardless of age. These patients are now explicitly eligible from 18 years upwards.
- Vaccinate before starting targeted therapy wherever possible. Aim for administration at least 14 days prior to initiation (1 month if feasible). The second Shingrix dose can be given after targeted therapy initiation, 8 weeks–6 months after the first dose.
- Do not allow equivocal serology to delay vaccination. VZV antibody titres are less reliable in immunosuppressed patients. Shingrix is safe and immunogenic irrespective of baseline serostatus. In borderline cases, err on the side of vaccinating.
- Consider Shingrix pragmatically in seronegative patients with urgent need for immunosuppression. Although not licensed for primary varicella prevention, Shingrix targets glycoprotein E and avoids the risks of live-attenuated vaccination in this setting.
- Communicate explicitly with primary care. Clinic letters should clearly state if patients meet criteria and require vaccination rather than implying this indirectly.

## Conclusion

Guidelines are necessary but do not, in themselves, guarantee effective implementation. The gap between recommendation and practice for shingles vaccination in severely immunosuppressed patients reflects competing priorities, fragmented responsibility between primary and secondary care and system-wide capacity constraints within rheumatology services.

Bridging this gap will require more than updated national documents. It demands practical, system-level solutions: embedding prompts into clinic workflows, explicit communication of eligibility to GPs, shared care protocols defining responsibilities and recognition that preventive interventions—however ‘peripheral’ they may seem—need dedicated attention if patients on targeted therapies are to be adequately protected from VZV reactivation.

## Data Availability

Data are available upon reasonable request.
